# Prof. Sanford B. Hunt

**Published:** 1854-11

**Authors:** 


### PROF. SANFORD B. HUNT.

We are glad to see that Dr. SANFORD B. HUNT, junior editor of the *Buffalo Medical Journal*, has been elected professor of Anatomy in the University of Buffalo. Dr. HUNT gives promise of attaining to great eminence in his profession, and the trustees of the Medical School of Buffalo have shown by his appointment a just appreciation of talents and industry. Among all the medical editors of our country we are not acquainted with one more laborious or judicious than Dr. HUNT, and in addition to his numerous contributions to his own Journal, he has been one of the correspondents of *Putnam's Magazine.*

